# Supplementary material for: Quasiparticle tunnel electroresistance in superconducting junctions
Source: Nat Commun. 2020 Jan 31;11:658. doi: 10.1038/s41467-020-14379-w (PMC6994500; doi:10.1038/s41467-020-14379-w)
Supplement: Supplementary file 1 — Supplementary Information [file 41467_2020_14379_MOESM1_ESM.pdf]

SUPPLEMENTARY INFORMATION FOR

"Quasiparticle Tunnel Electroresistance in Superconducting Junctions"

V. Rouco[1], R. El Hage[1] , A. Sander[1], J. Grandal[2], K. Seurre[1], X. Palermo[1], J. Briatico[1], S. Collin[1], J. Trastoy[1], K. Bouzehouane[1], A.I. Buzdin[3], G. Singh[4], N. Bergeal[4], C. Feuillet-Palma[4], J. Lesueur[4], C. Leon[2], M. Varela[2], J. Santamaría[1,2] and Javier E. Villegas[1]

[1]*Unité Mixte de Physique, CNRS Thales, Université Paris-Sud, Université Paris Saclay, 91767 Palaiseau, France*

[2]*Grupo de Física de Materiales Complejos, Dpt. Física de Materiales, Universidad Complutense de Madrid, 28040 Madrid, Spain*

[3]*Univ Bordeaux, LOMA UMR CNRS 5798, F-33405 Talence, France*

[4]*Laboratoire de Physique et d'Etude des Matériaux, ESPCI Paris, Université PSL, CNRS, 75005 Paris (France)*

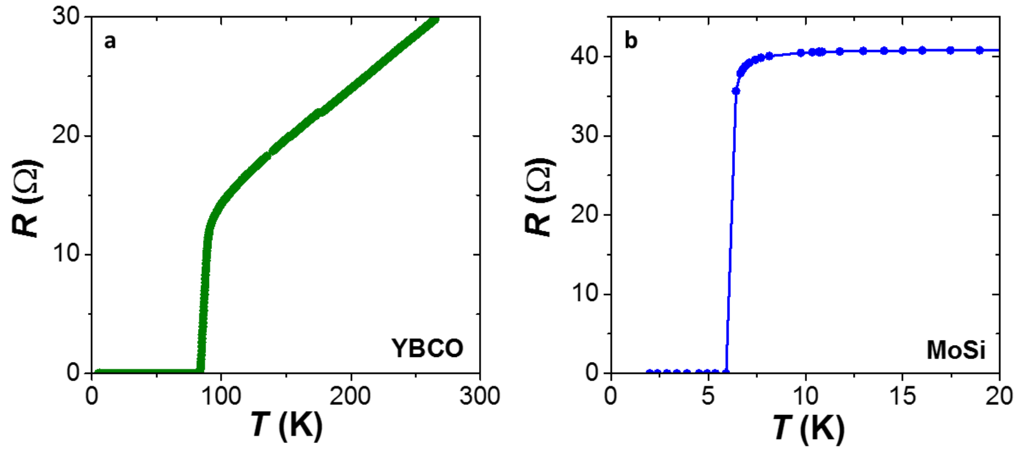

Supplementary Figure 1: Superconducting properties of the electrodes. 4-probe resistance measurement of (a) YBCO and (b) MoSi electrodes, measured with a current *I* = 10 μA. The onset of the superconducting transition is observed at T_C ~91 K for YBCO and T_C ~ 6.8K for MoSi**.**

Material	Reduction reaction	Reduction potential E_0 (V)
YBCO	$Cu^{+3} + e^{+} \rightarrow Cu^{+2}$	2.4
BFO	$Fe^{+3} + e^{+} \rightarrow Fe^{+2}$	0.7
$Mo_{80}Si_{20}$	$Mo^{+3} + 3e^{-} \rightarrow Mo$	-0.2
$Mo_{80}Si_{20}$	$Si^{+2} + 2e^{-} \rightarrow Si$	-0.8
ITO (In_2O_3 98%/SnO_2 2%)	In^{+3}+ +e - $\rightarrow$ In+2	-0.49

Supplementary Table 1: Reduction potentials of the junction materials. Obtained from the *Table of Standard Electrode Potentials* by Milazzo et al., edited by Willey (Chichester), 1978. For YBCO and BFO, we have considered the change of valence of Cu and Fe expected[1,2] when oxygen is removed from the structure. For MoSi we quote the two elements Mo and Si, and for ITO we select In since In_2O_3 constitutes 98% (weight) of the material. Data for $SrTiO_3$ not available as the expected reduction Ti^{+4} + e - $\rightarrow Ti^{+3}$ is not tabulated. We see from the table that YBCO has the highest reduction potential. Thus, the redox reaction through which oxygen is transferred into the counter-electrode (MoSi or ITO) is expectedly spontaneous. The voltage required to reverse this reaction equals ΔE_0, which lies between 2.6 and 3.2 V in all cases.

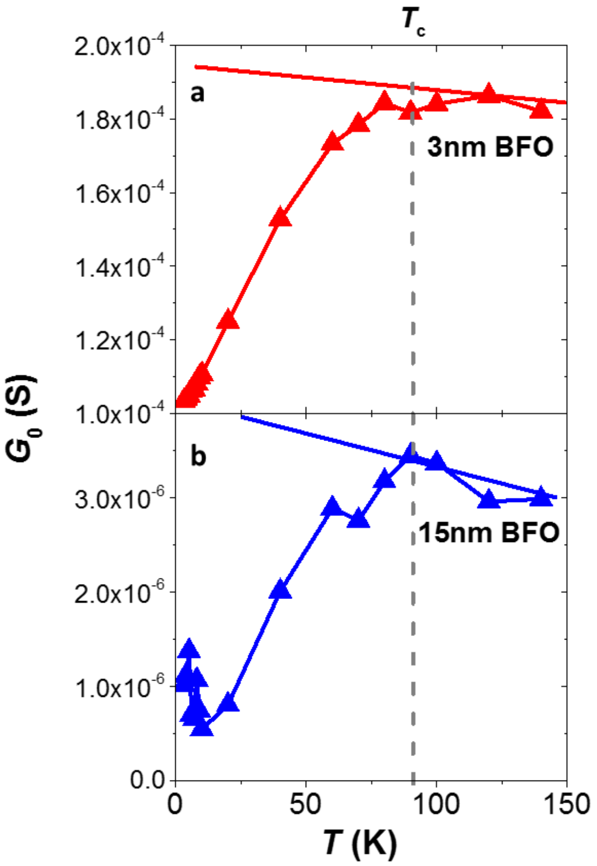

Supplementary Figure 2: Zero bias conductance vs. temperature in the ON state. G_0 data shown in Figs. 2h and 2i, here displayed in linear scale to enable appreciation of the temperature at which $G_0(T)$ departs from the high-temperature trend indicated by the straight lines. The temperature is approximately the T_C of optimally doped YBCO, as pointed by the vertical dashed line.

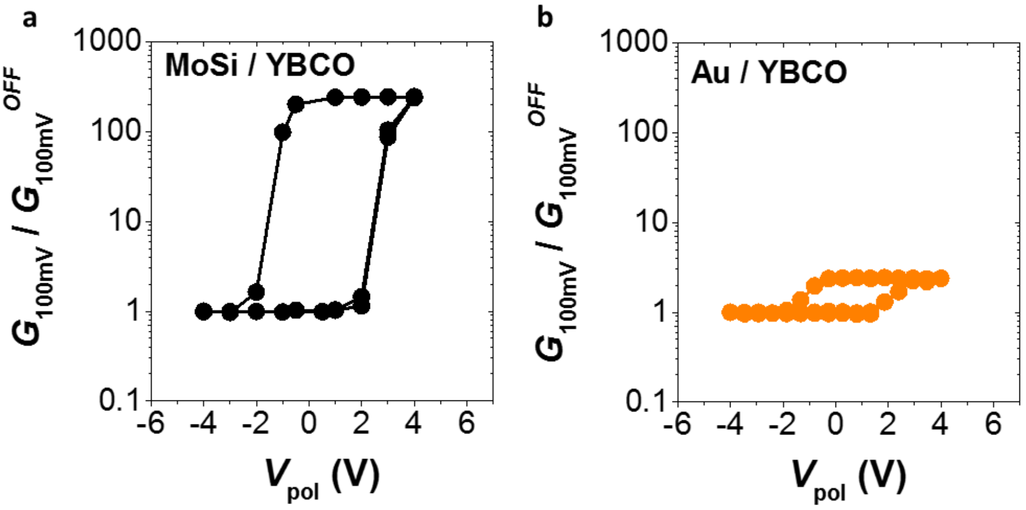

Supplementary Figure 3: Conductance switching for direct contacts: comparison between YBCO/MoSi and YBCO/Au junctions. Conductance switching measured at 100 mV in (a) a MoSi/YBCO junction and in (b) Au/YBCO junction of the same area (200µm^2). One can see that the conductance changes more than two orders of magnitude in the first case, but only a factor of ~2 in the second, as expected from earlier experiments in Au/YBCO contacts[3]. This very different behaviour demonstrates that a metal with tendency to oxidize (MoSi) is required to obtain large conductance switching comparable with the TER of ferroelectric tunnel junctions.

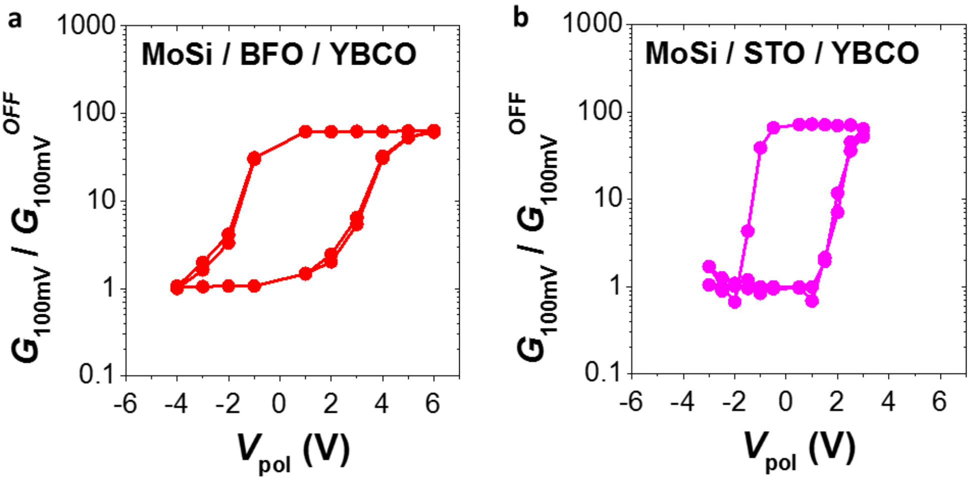

Supplementary Figure 4: Conductance switching for junctions with different barriers: comparison between ferroelectric (BFO) and non-ferroelectric (STO) barriers. Conductance switching measured at 100 mV in (a) a MoSi/BFO_{3nm}/YBCO junction and in (b) MoSi/STO_{3nm}/YBCO junction of the same area (200µm^2). $SrTiO_3$ is a non-ferroelectric band insulator. One can see that the conductance switching is qualitatively and quantitatively similar in both types of junctions, which shows that the ferroelectric character of BFO is not playing a major role in the conductance switching behaviour.

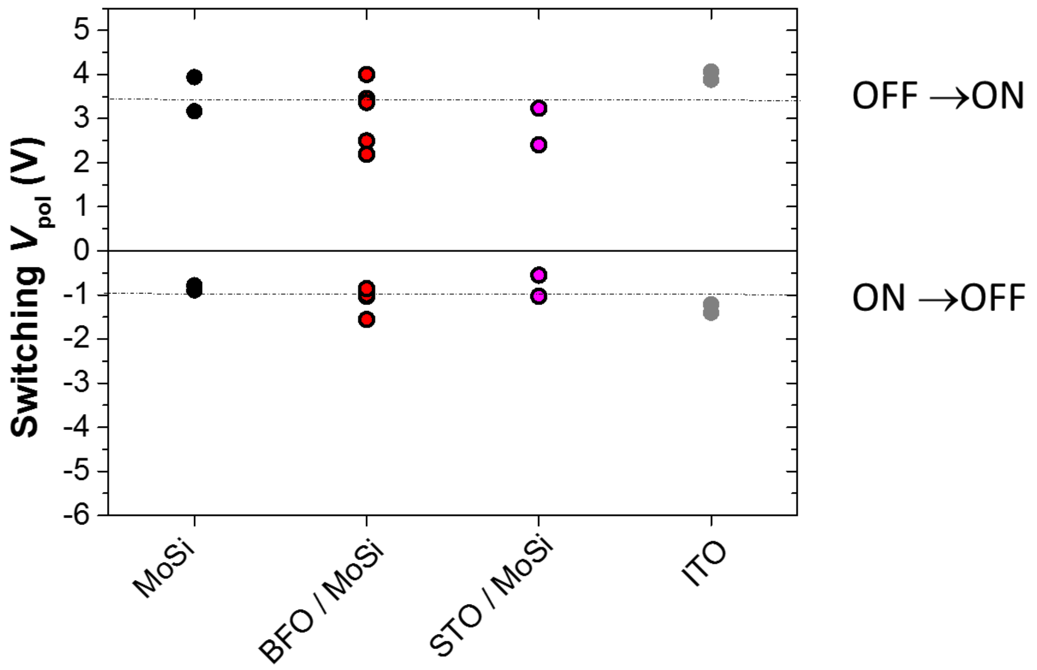

Supplementary Figure 5: Switching voltages for different types of junctions. Voltages V_{pol} for switching from the OFF into the ON state ($V_{\text{pol}} > 0$) and vice versa ($V_{\text{pol}} > 0$) for different types of junctions in which the conductance switching size is similar (around two orders of magnitude at 100 mV). The different types of junctions include direct contacts between $Mo_{80}Si_{20}$ (MoSi) or In_2O_3/SnO_2 (ITO) and YBCO, as well as junctions in which an interlayer is placed in between MoSi and YBCO, particularly $BiFeO_3$ (BFO) and $SrTiO_3$ (STO). For all these junctions, the switching voltages are defined as the voltage for which $\left(G_{100\text{mV}} - G_{100\text{mV}}{}^{OFF}\right)/G_{100\text{mV}}{}^{ON}$ = 0.5. One can see that, within the experimental error given by the data scattering for each type of junction (several devices were measured in each case), the switching V_{pol} are rather similar for all junctions. Notice the asymmetry, with V_{pol} (ON→OFF)~-1V and V_{pol} (ON→OFF) ~ 3.5 V. The latter is similar to the difference between reduction potential of the junction electrodes, ΔE_0, as calculated in section 2 above.

Supplementary Note 1: Estimates of tunnelling barrier height and width from fits to BDR model.

In order to estimate the tunnel junction parameters, we fitted the conductance curves $G(V_{BIAS})$ in the normal state (T = 90K) to the Brinkman, Dynes, and Rowell (BDR) model[7].

$$\frac{G(V)}{G(0)} = 1 - \left(\frac{A_0 \Delta\varphi}{16\varphi^{\frac{3}{2}}}\right) eV + \left(\frac{9}{128}\frac{A_0{}^2}{\varphi}\right)(eV)^2 \qquad (1)$$

where $\Delta\varphi$ is the barrier asymmetry, φ is the average barrier height, and $A_0 = 4(2m)^{1/2}d/3\hbar$ with m the electron mass and d the tunnel barrier thickness.

We have only studied the curves in the OFF state at low bias (< 100 mV), where the conductance shows a parabolic dependence. Fitting of the ON states curves to this model is not possible since the nearly linear conductance background $G \propto |V_{BIAS}|$ indicates the predominance of inelastic tunneling[4].

The fit shown for 0 nm YBCO yields an energy barrier 0.15 ± 0.02 eV, a total barrier thickness 5.0 ± 0.2 nm, and a barrier asymmetry ~ 20% of the barrier height. The fit for the 3 nm BFO film is for a barrier width 8.0 ± 0.5 nm, in consistency with the above result for 0 nm BFO. That barrier width implies a barrier height 0.53±0.03 eV.

We show in Supplementary Figure 7 a scheme of the tunnel barrier as deduced from the above fits.

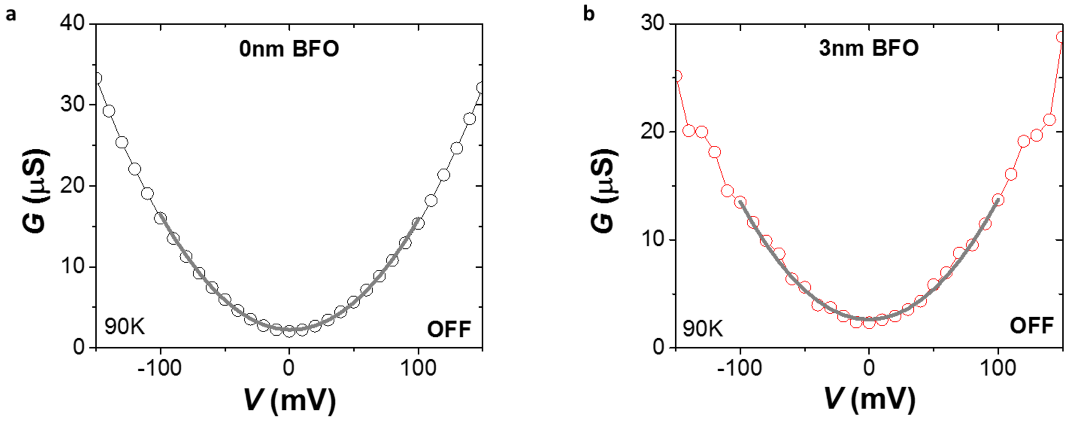

Supplementary Figure 6: Fits of the normal-state tunnelling conductance to the BDR model for junctions with 0 nm BFO and 3 nm BFO

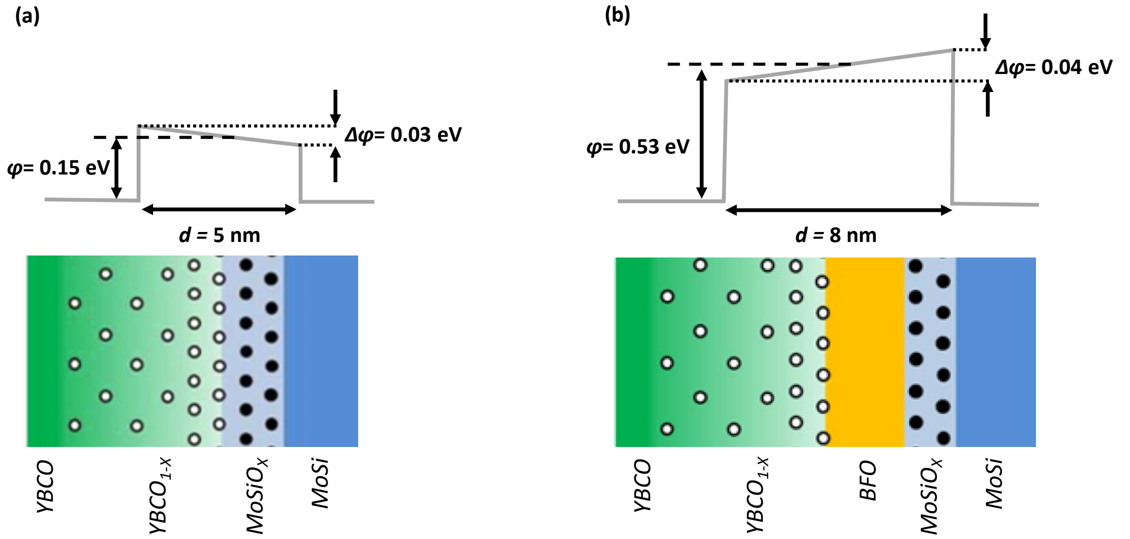

Supplementary Figure 7: Scheme of the energy barrier across the tunnel junction without BFO **(a)** and with **(b)** 3 nm BFO.

Supplementary Note 2: Estimates of the barrier strength through simulations with the BTK theory.

Besides drastically varying in the junctions' resistance, the switching between the ON and OFF states produces spectral changes in the differential conductance $g(V)$. These changes can be analysed in the frame of the Blonder-Tinkham-Klapwijk (BTK)[7] theory for superconductor/metal junctions, which explains the conductance features associated to the superconducting gap(Δ) through a parameter Z that measures the junction's barrier strength (the higher Z, the lower the junction transparency).

The insets of Supplementary Figure 8 (a) and (b) respectively show the normalized conductance $g_S(V)/g_N(V)$ in the ON and OFF state, with $g_S(V)$ measured in the superconducting state (at T=3.2 K) and $g_N(V)$ measured just above T_C. One can see that the gap-related "dip" around zero bias is deeper in the OFF than in the ON state. Within the BTK theory, this can be interpreted as Z being higher in the OFF that in the ON state, which is consistent with the junction resistance being much higher in the OFF that in the ON state.

We carried out a more quantitative analysis using the BTK theory extended to the case of c-axis tunnelling into d-wave superconductors[8,9]. Because the BTK theory does not account for the temperature and bias dependence of the background conductance (evident in the experiments for $eV>>\Delta$), comparison between the BTK conductance and the experiments requires removing the (weak) quadratic background highlighted by the magenta dotted lines in the insets of Supplementary Figure 8. For this, we re-normalize $g_S(V)/g_N(V)$ to the background, which yields the data shown in the main panel of Supplementary Figure 8 (black dots). These data are to be compared to the simulations made with the BTK theory (coloured lines).

We fist attempted fitting the bare BTK theory[8,9] to the experimental data, by fixing the value of Δ according to the analysis in Fig. 4 and varying Z. Examples of the calculated BTK conductance are depicted with blue lines in Supplementary Figure 8 (a) and (b). One can see that the experimental curves show a strong smearing of coherence peaks near the gap edge as compared with the bare BTK theory. In order to improve the fits, we considered broadening effects, in particular the effect of finite quasiparticle lifetime[10,11] (which can be quantified by means of a phenomenological parameter Γ) as well as the presence of inhomogeneity in the properties of the superconductor[12]. The red solid lines show a simulations including those effects, particularly by considering[10,11] Γ=3 mV and averaging over 50 simulations with a standard deviation of the gap of σ_Δ=5 mV in order to simulate the effect of inhomogeneity. One can see that this allows a better fit to the experimental results.

Besides providing further support to the interpretation of the tunnelling conductance spectra given in the main text, the above analysis provides a solid estimate of the barrier strength Z in the ON and OFF states, respectively Z_{ON}~3 and Z_{OFF}~30.

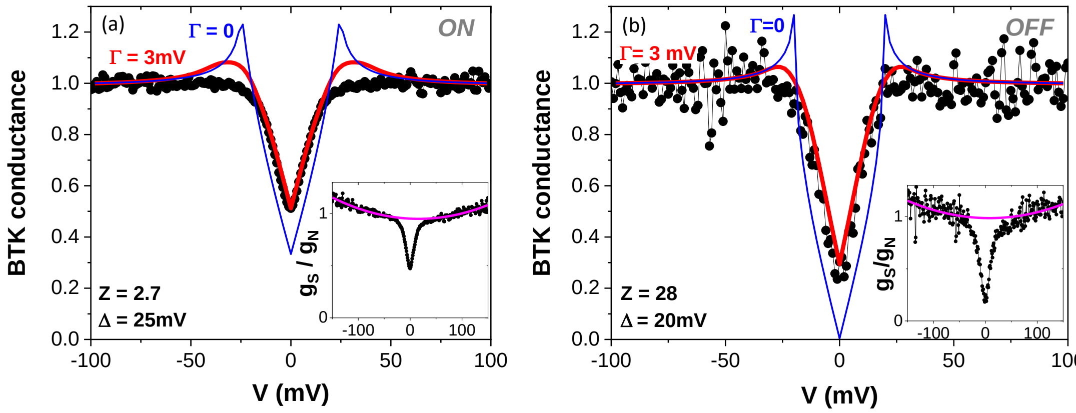

Supplementary Figure 8: Analysis of the conductance using the BTK theory. For a 3nm BFO sample. In the ON state (a) in the OFF one (b). Experimental data are depicted by black dots and theoretical simulations by lines (blue and red). The values for the parameters Z and Δ, and the different values of inelastic scattering parameter Γ are indicated in the legend. of Insets: show the conductance in the superconducting state g_S(measured at 3.2 K) normalized to the normal-state conductance g_N (measured right above T_C, at T=90 K in the ON state and T=70 K in the OFF state). The weak background dependence highlighted by the pink dotted line is obtained from a fit of $g_S(V)/g_N(V)$ to a quadratic polynomial in the range 30mV<$|V|$<100. mV), and that is removed for the BTK analysis in the main panel.

Supplementary Note 3: Modelling of the temperature dependent tunnelling electro-resistance via BTK theory.

The simulations of differential conductance *vs.* bias at different temperatures shown in Fig. 5 were calculated with the generalized BTK theory[8,9], in which temperature effects are included through the Fermi-Dirac function and the temperature dependence of superconducting gap $\Delta(T) = \Delta_0 \tanh\left(2.06\sqrt{T_C/T - 1}\right)$ generally considered for d-wave superconductors[13]. We performed calculations both for the ON and OFF states [see examples in Fig. 5a using the parameters obtained in Supplementary Note 3 (we assume that Γ is temperature independent as elsewhere[13]). From the calculations, we extracted the BTK conductance at zero-bias (G_0) and at 100 mV (G_{100}) as a function of temperature, which is shown in Fig. 5b. Note that for clarity we have multiplied the BTK conductance by a factor G_{100} that is different in the ON and OFF, thus mimicking the ON/OFF conductance switching. Notice finally that, as discussed in Supplementary Note 3, the BTK theory does not account for the temperature and bias dependence of the background conductance. Consequently, although the BTK simulations reproduce the main features of the bias and temperature dependent ER, they do not account for the temperature dependent G_{100} observed experimentally, and cannot perfectly reproduce the experimental ER_0/ER_{100} vs. T.

Supplementary references

1. Temmerman, W. M., Winter, H., Szotek, Z. & Svane, A. Cu valency change induced by O doping in YBCO. *Phys. Rev. Lett.***86**, 2435–2438 (2001).

2. Sæterli, R., Selbach, S. M., Ravindran, P., Grande, T. & Holmestad, R. Electronic structure of multiferroic BiFeO3 and related compounds: Electron energy loss spectroscopy and density functional study. *Phys. Rev. B - Condens. Matter Mater. Phys.***82**, 29–33 (2010).

3. Plecenik, A. *et al.* Influence of bias voltage history on conductance properties of YBaCuO/normal metal junctions. *Phys. C Supercond. its Appl.***301**, 234–242 (1998).

4. Kirtley, J., Washburn, S. & Scalapino, D. Origin of the linear tunneling conductance background. *Phys. Rev. B. Condens. Matter***45**, 336–346 (1992).

5. Boyn, S. *et al.* Engineering ferroelectric tunnel junctions through potential profile shaping. *APL Mater.***3**, (2015).

6. Tzung-Lin Li *et al.* Novel dual-metal gate technology using Mo-MoSi/sub x/ combination. *IEEE Trans. Electron Devices***53**, 1420–1426 (2006).

7. Blonder, G. E., Tinkham, M. & Klapwijk, T. M. Transition from metallic to tunneling regimes in superconducting microconstrictions: Excess current, charge imbalance, and supercurrent conversion. *Phys. Rev. B***25**, 4515–4532 (1982).

8. Kashiwaya, S., Tanaka, Y., Koyanagi, M., Takashima, H. & Kajimura, K. Origin of zero-bias conductance peaks in high-Tc superconductors. *Phys. Rev. B***51**, 1350 (1995).

9. Wei, J. Y. T., Yeh, N. C., Garrigus, D. F. & Strasik, M. Directional tunneling and andreev reflection on YBa2Cu3O7 − δ single crystals: Predominance of d-wave pairing symmetry verified with the generalized blonder, tinkham, and klapwijk theory. *Phys. Rev. Lett.***81**, 2542–2545 (1998).

10. Dynes, R. C., Narayanamurti, V. & Garno, J. P. Direct Measurement of Quasiparticle-Lifetime Broadening in a Strong-Coupled Superconductor. *Phys. Rev. Lett.***41**, 1509–1512 (1978).

11. Pleceník, A., Grajcar, M., Beňačka, Š., Seidel, P. & Pfuch, A. Finite-quasiparticle-lifetime effects in the differential conductance of Bi 2 Sr 2 CaCu 2 O y /Au junctions. *Phys. Rev. B***49**, 10016–10019 (1994).

12. Feigel'man, M. V. & Skvortsov, M. A. Universal Broadening of the Bardeen-Cooper-Schrieffer Coherence Peak of Disordered Superconducting Films. *Phys. Rev. Lett.***109**, 147002 (2012).

13. Park, W. K., Greene, L. H., Sarrao, J. L. & Thompson, J. D. Andreev reflection at the normal-metal/heavy-fermion superconductor Ce Co In 5 interface. *Phys. Rev. B***72**, 052509 (2005).
